# Hindbrain rhombomere centers harbor a heterogenous population of dividing progenitors which rely on Notch signaling

**DOI:** 10.3389/fcell.2023.1268631

**Published:** 2023-11-02

**Authors:** Carla Belmonte-Mateos, Lydvina Meister, Cristina Pujades

**Affiliations:** Department of Medicine and Life Sciences, Universitat Pompeu Fabra, Barcelona, Spain

**Keywords:** hindbrain rhombomeres, zebrafish, neurogenesis, neural progenitors, Notch signaling, cell division mode

## Abstract

Tissue growth and morphogenesis are interrelated processes, whose tight coordination is essential for the production of different cell fates and the timely precise allocation of stem cell capacities. The zebrafish embryonic brainstem, the hindbrain, exemplifies such coupling between spatiotemporal cell diversity acquisition and tissue growth as the neurogenic commitment is differentially distributed over time. Here, we combined cell lineage and *in vivo* imaging approaches to reveal the emergence of specific cell population properties within the rhombomeres. We studied the molecular identity of hindbrain rhombomere centers and showed that they harbor different progenitor capacities that change over time. By clonal analysis, we revealed that cells within the center of rhombomeres decrease the proliferative capacity to remain mainly in the G1 phase. Proliferating progenitors give rise to neurons by asymmetric and symmetric neurogenic divisions while maintaining the pool of progenitors. The proliferative capacity of these cells differs from their neighbors, and they are delayed in the onset of Notch activity. Through functional studies, we demonstrated that they rely on Notch3 signaling to be maintained as non-committed progenitors. In this study, we show that cells in rhombomere centers, despite the neurogenic asynchrony, might share steps of a similar program with the rhombomere counterparts, to ensure proper tissue growth.

## Introduction

The generation of brain cell diversity occurs concomitantly with tissue morphogenesis, resulting in changes in the position of neuronal progenitors and their derivatives over time. Thus, one of the main unsolved questions is how multiple cell types are generated and maintained in highly organized spatial patterns upon morphogenesis and how changes in this ground plan can result in pathologies. The hindbrain has been proven to be a good experimental system to address the bases of neuronal diversity since it displays stereotypic growth dynamics while undergoing tissue segmentation along the anteroposterior (AP) axis (Krumlauf and Wilkinson, 2021) and lumen formation (Lowery and Sive, 2004; Gutzman and Sive, 2010).

Hindbrain segmentation results in rhombomeres that constitute developmental units of gene expression and cell lineage compartments (Fraser et al., 1990; Lumsden, 2004; Jimenez-Guri et al., 2010; Krumlauf and Wilkinson, 2021), separated by interhombomeric boundaries. During the last few years, it has been demonstrated that neurogenic capacities are sequentially allocated along the AP axis (Nikolaou et al., 2009; Esain et al., 2010; Gonzalez-Quevedo et al., 2010), with boundary cells engaging later than rhombomeres in neurogenesis (Peretz et al., 2016; Voltes et al., 2019; Pujades, 2020; Hevia et al., 2022). During the first neurogenic phase, hindbrain boundaries function as an elastic mesh to restrict cell intermingling between adjacent cell lineages (Calzolari et al., 2014; Letelier et al., 2018). Boundary cells undergo mechanical tension that activates the YAP/TAZ pathway that maintains them in the proliferative progenitor state (Voltes et al., 2019; Engel-Pizcueta and Pujades, 2021). Moreover, no synchronous patterning of neurogenesis is observed within the very same rhombomeres. Neurogenesis becomes confined to the cell population adjacent to rhombomere boundaries (Nikolaou et al., 2009), and the segment centers comprise a non-neurogenic progenitor population with a different molecular identity (Esain et al., 2010; Gonzalez-Quevedo et al., 2010; Tambalo et al., 2020), which signals through FGF to instruct neuronal organization (Gonzalez-Quevedo et al., 2010). Although FGF signaling is not essential for the survival or maintenance of hindbrain neural progenitors, it controls their fate by coordinately regulating *sox9* and *oligo2* (Esain et al., 2010). However, little is known about how these cells are maintained as progenitors.

Thus, in this study, we aimed at understanding how the neurogenic capacity was asynchronously distributed within the rhombomeres. We have explored their molecular identity and their proliferative capacity. Clonal analyses and cell proliferation experiments revealed that cells within the rhombomere centers are held as slow-dividing progenitors. Multicolor cell lineage analysis allowed us to demonstrate that rhombomere centers harbor progenitor cells that can engage in neurogenesis, contributing to the neuronal lineage. Functional analyses revealed the role of Notch3 signaling in the control of these cells as non-committed progenitors during hindbrain morphogenesis.

## Materials and methods

### Ethics declarations and approval for animal experiments

All procedures were approved by the institutional animal care and use ethics committee (Comitè Etica en Experimentació Animal, PRBB) and the Generalitat of Catalonia (Departament de Territori i Sostenibilitat), and implemented according to the National and European regulations. Government and University veterinary inspectors examined the animal facilities and procedures to ensure that animal regulations were correctly followed. The PRBB animal house holds the AAALAC International approval B9900073. All the members entering the animal house have to hold international FELASA accreditation. The Project License covering the proposed work (Ref 10642, GC) pays particular attention to the 3Rs.

### Zebrafish strains

Embryos were obtained by mating adult fish using standard methods. All zebrafish strains were maintained individually as inbred lines. Tg[elA:GFP] (Labalette et al., 2011) and Mu4127 (Distel et al., 2009) transgenic lines were used as landmarks of rhombomeres 3 and 5, displaying GFP and mCherry, respectively. Tg[BCP:H2AmCherry] and Tg[BCP:H2B-GFP] were used as landmarks of hindbrain boundaries (Hevia et al., 2022). Tg[nestin:GFP] carries GFP at 3.9 Kb upstream of the nestin promoter and labels neural progenitors (Lam et al., 2009). The Tg[HuC:GFP] line was used to label the whole neuronal differentiated domain (Park et al., 2000). The dual Fucci transgene, Tg[Fucci], ubiquitously produces both a Cerulean-tagged degron, which is detectable during the S/G2/M phases of the cell cycle, and a Cherry-tagged degron, which is only detectable during the G1 phase (Bouldin and Kimelman, 2014). The readout of the Notch-activity line Tg[tp1:d2GFP] was constructed using the *tp1* promoter (Clark et al., 2012; Hevia et al., 2022), and Notch-active cells express destabilized GFP. The *notch3* ^*fh332*^ null allele originated from ENU-induced nonsense mutation and was identified by genotyping genomic DNA from fin clips or embryonic tails, according to Alunni et al. (2013). Embryos homozygous for *notch3* ^*fh332/fh332*^ were obtained by in-cross of heterozygous carriers. As controls, both *notch3* ^*+/+*^ and *notch3* ^*fh332/+*^ embryos were used since heterozygous embryos displayed the wild-type phenotype. The *hey1* ^*ha11*^ mutant allele harbors a 11-bp deletion, causing a frameshift leading to the production of a truncated protein; its presence was identified by genotyping genomic DNA from fin clips or embryonic tails, according to Than-Trong et al. (2018). Embryos homozygous for *hey1* ^*ha11/ha11*^ were obtained by in-cross of heterozygous carriers. As controls, both *hey* ^*+/+*^ and *hey1* ^*ha11/+*^ embryos were used since heterozygous embryos displayed the wild-type phenotype.

### Confocal imaging of whole-mount embryos

Anesthetized live embryos expressing genetically encoded fluorescence and stained fixed samples were mounted in 1% or 0.8% low melting point (LMP) agarose with the hindbrain positioned toward the glass bottom of Petri dishes (MatTek) to achieve dorsal views of the hindbrain. Imaging was performed under an SP8 Leica confocal microscope.

### Whole-mount *in situ* hybridization

Embryo whole-mount *in situ* hybridization was adapted from the study by Thisse and Thisse (2008). The following riboprobes were generated by *in vitro* transcription from cloned cDNAs: *ascl1b* (Allende and Weinberg, 1994), *erm* (Riley et al., 2004), *meteorin* and *meteorin-like* (Tambalo et al., 2020), *neurod4* (Park et al., 2003), *neurog1* (Itoh and Chitnis, 2001), and *notch1b* (Dyer et al., 2014). The other probes were generated by PCR amplification by adding the T7 or Sp6 promoter sequence in the Rv primers: *fabp7a* Fw: 5’ –GAC TGA ACT CAG CGA CTG TAC– 3′ and Rv: 5’ –AGG CCT CAA TAA TAC ACT CCC–T7 3’; *hey1* Fw: 5’ –GCA GAG ACT GCA CGT TAC CTC– 3′ and Rv: 5’ –GCC CCT ATT TCC ATG CTC CAG–T7 3’; *notch1a* Fw: 5’ –ACT TCG AAA TCG CTC ATC– 3′ and Rv: 5’ –TCT TCC TGG AGA CGA CCA C–T7 3’; *notch3* Fw: 5’ –ATG GGG AAT TAC AGC CTT TG– 3′ and Rv: 5’ –GGC AAA CAA GCA ATT CGT A–SP6 3’; *slc1a2a* Fw: 5’–GGG AAA GAT GGG AGA GAA GG– 3′ and Rv: 5’ –AGG ACT GTG TCT TGG CCA TC–T7 3’; *sox9b* Fw: 5’ –GGG CTG AAG ATG AGT GTG TC– 3′ and Rv: 5’ –CTT CAG ATC CGC TTA CTG CAC–T7 3’. For fluorescent *in situ* hybridization, FLUO- and DIG-labeled probes were detected with TSA fluorescein and Cy3, respectively.

### *In toto* embryo immunostaining

Embryos were blocked in 10% neutralized goat serum and 2% bovine serum albumin (BSA) in PBST for 2 h at RT, except after *in situ* hybridization, when they were blocked in 5% neutralized goat serum in PBS-Tween 20 (PBST) for 1 h. They were then incubated O/N at 4°C with the corresponding primary antibodies: anti-DsRed (1:500; Clontech), mouse anti-GFP ([1:100]; Thermo Fisher), anti-fabp7a ([1:100], Millipore), rabbit anti-GFP ([1:400], Torrey Pines), rabbit anti-pH3 (1:200; Upstate), rabbit anti-Sox2 ([1:100], Abcam), and mouse anti-HuC ([1:100], Thermo Fisher) antibodies. After extensive washing with PBST, embryos were incubated with secondary antibodies conjugated with Alexa Fluor^®^488, Alexa Fluor^®^594, or Alexa Fluor^®^633 ([1:250], Invitrogen). DAPI ([1:5000], Molecular Probes) was used to label cell nuclei.

### Cell proliferation

#### EdU incorporation experiments

Cells in the S phase were detected by EdU incorporation using the Click-It™ EdU Alexa Fluor™ 647 Imaging Kit (C10340, Thermo Fisher Scientific), following the supplier instructions with some modifications. Briefly, embryos were dechorionated, incubated in 500 µM EdU, diluted in 7% DMSO, and placed on ice during the first hour for better EdU incorporation. Afterward, they were either washed three times with an embryo medium to wash out EdU or fixed in 4% PFA for 4 h at RT and dehydrated in MetOH. After progressive rehydration, embryos were permeabilized with 10 mg/mL Proteinase K (Invitrogen) for 35 min, post-fixed 40 min in 4% PFA, and washed in PBT. Embryos were then incubated for 1 h in 1% DMSO/1% Triton X-100/PBS. The Click-It reaction was carried out according to the manufacturer’s instructions for 60 min at RT. Embryos were washed in PBT and then used for *in situ* hybridization with *fabp7a*. To determine the position of boundaries and, therefore, rhombomere centers, we used the Tg[BCP:H2B-GFP] line, and GFP was revealed by immunostaining. Imaging was undertaken with the Airyscan2 LSM 980 microscope using a ×20 dry objective.

#### Cell cycle phase dynamics

In order to follow the cell cycle dynamics, the distribution of PCNA-GFP in the cell nuclei was assessed at different cell cycle phases. Mu4127 embryos, as landmarks of r3 and r5, were injected between the 16-cell stage and the 32-cell stage with PCNA-GFP mRNA, let to grow until the desired stage, and in vivo imaged under the Airyscan2 LSM980 microscope using ×40 (1.2NA) glycerol immersive objectives with Zeiss acquisition software. Samples were maintained at 28°C while being imaged. Optical sections were acquired through the entire hindbrain volume from dorsal to ventral sides (z = 1 μm). For quantification, we counted the S-phase cells and the total number of PCNA-labeled nuclei in the delimitated areas (76 µm × 7 µm) (Figure 3B, see colored framed regions), both in the centers of rhombomeres 3, 4, and 5 and in the corresponding boundary-flanking regions (n = 24 regions; N = 4 embryos). We then calculated the ratio between the number of nuclei in the S phase and the total number of PCNA-GFP-labeled nuclei at 42 and 48 hpf. Finally, the percentage of S-phase cells, both in boundary-flanking regions and rhombomere centers, was calculated for further comparisons.

### Zebrabow multicolor cell clonal analysis

For multicolor cell clonal analyses, Tg[HuC:GFP] embryos were injected with the hsp:ZEBRABOW construct (Brockway et al., 2019) between the 8-cell stage and the 16-cell stage. Embryos were heat-shocked for 1 h at 37°C just 3 hours before imaging. Then, they were washed in a fresh embryo medium and maintained at 28°C until the desired imaging time. The position of the rhombomeric centers was achieved by morphological repairs, thanks to the fact that the HuC domain displays a wide ventral U-shape with enrichment in the mantle zone of boundaries (Belzunce et al., 2020). Images were acquired with the Leica SP8 confocal microscope using a ×20 water immersion objective with zoom ranging from 1.0 to 1.5. Embryos were then incubated at 28°C for 12 h and reimaged with the same settings.

### TUNEL analyses

The distribution of apoptotic cells was determined by TdT-mediated dUTP nick-end labeling of the fragmented DNA (TUNEL, Roche). Briefly, embryos were fixed in 4% PFA, dehydrated in 100% MetOH, permeabilized with 80% acetone/H_2_0, and preincubated with the TUNEL mixture for 3 h at 37°C, according to the manufacturer’s instructions. DAPI ([1:5000], Molecular Probes) was used to label nuclei.

### Pharmacological treatments

Embryos were treated either with 10 μM of the gamma-secretase inhibitor LY411575 (Sigma-Aldrich) or DMSO for control. The treatment was applied to fish water at 28.5°C for 6 h at the indicated stages. After treatment, embryos were fixed in 4% PFA for further analysis.

### Sox2 and HuC volume quantifications

For the quantification of the progenitor and neuronal differentiation volumes in rhombomere 4 (Supplementary Figure S3), we developed a macro in ImageJ that can be found at https://github.com/cristinapujades/Belmonte-Mateos-et-al-2023.

## Results

### Cells within the rhombomere centers display a specific progenitor–marker combination

**FIGURE 1:**
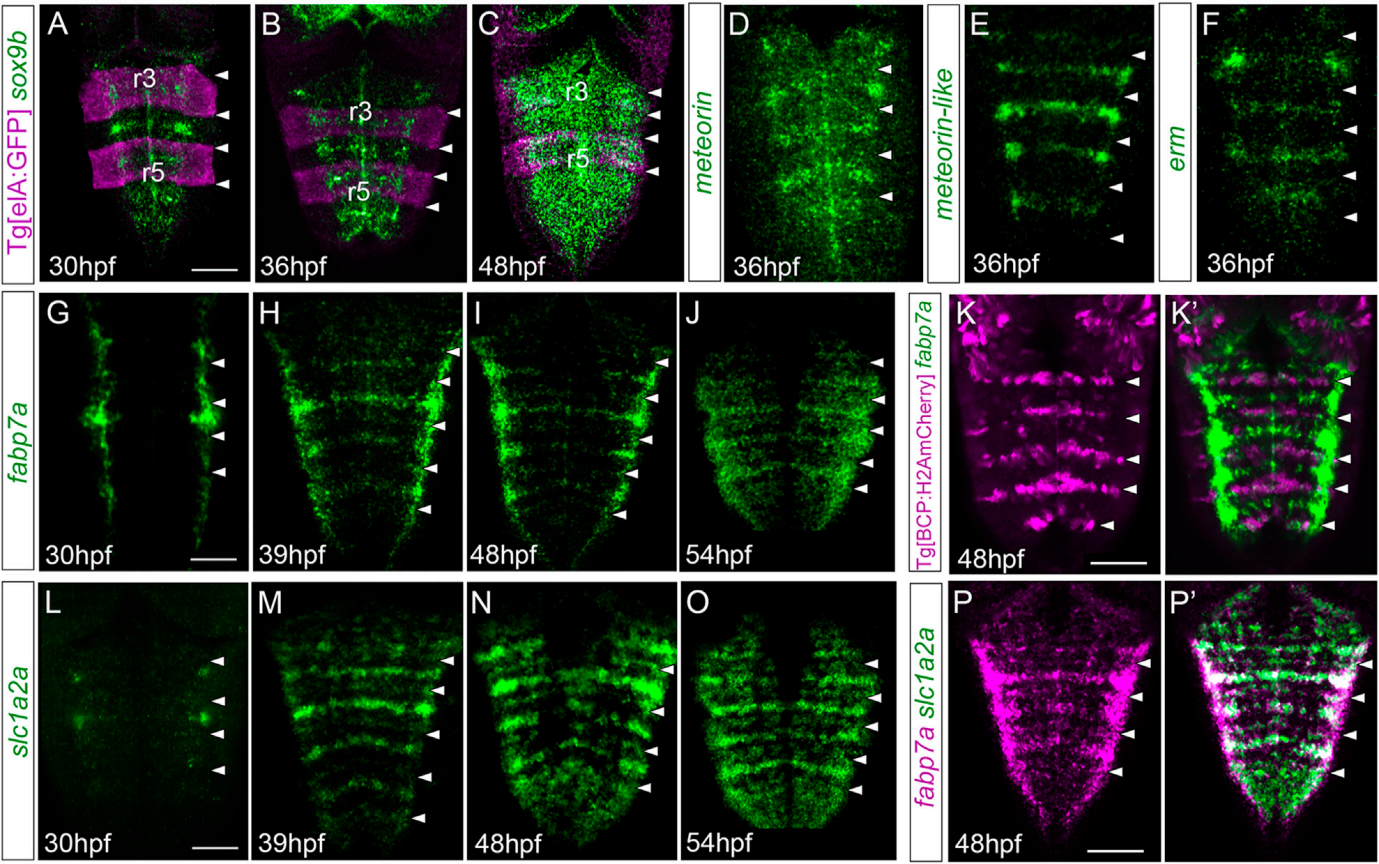
Hindbrain rhombomere centers display a specific combination of gene expression. **(A–C)** Tg[elA:GFP], **(D–J,L–P,P’)** wild type, or **(K,K’)** Tg[BCP:H2AmCherry] embryos at the indicated embryonic stages were *in situ* hybridized with *sox9* **(A–C)**, *meteorin* **(D)**, *meteorin-like* **(E)**, *erm* **(F)**, *fabp7a* **(G–K,K’,P,P**’**)**, and *slc1a2a* **(L–P, P’)**. Images are displayed as the single gene expression channel **(D–K, L–P)** or the overlay of the two corresponding channels **(A–C, K’, P’)**. All images are dorsal maximal intensity projections (MIP) with anterior to the top. White arrowheads indicate the rhombomeric boundaries. Note the enrichment of several markers in rhombomere centers. r3 and r5, rhombomeres 3 and 5. Scale bar, 50 μm.

To assess whether the putative differences in neurogenic capacity within the very same rhombomere could be foreshadowed by differential gene expression, we analyzed the expression of several well-characterized markers in embryonic hindbrains during a temporal period, encompassing the first and the second neurogenesis phases. To position the cells along the anteroposterior (AP) axis within the very same rhombomere, cells at the centers vs. cells at the neighboring regions, we resorted to zebrafish transgenic lines such as Mu4127 and Tg[elA:GFP], which label rhombomeres (r) 3 and 5 in mCherry and GFP, respectively (Distel et al., 2009; Labalette et al., 2011), and Tg[BCP:H2AmCherry/H2B-GFP] as landmarks of hindbrain boundaries (Hevia et al., 2022). First, we assessed the expression of *sox9b*, a transcription factor used as a marker of neural progenitors with radial glial features, which is necessary to maintain cells in the neural progenitor state but is not sufficient for the generation of glial cells (Stolt et al., 2003). From 30 hpf to 36 hpf, *sox9b* expression was enriched in rhombomere centers (Figures 1A, B). After 36 hpf, *sox9b* became homogenously expressed along the AP axis (Figure 1C). During this period, rhombomere centers displayed other well-described genes, such as *meteorin*, *meteorin-like*, and *erm,* the read-out of FGF-activity (Figures 1D–F). Next, we profiled hindbrains with markers such as *fabp7a* and *slc1a2a*, whose expression features radial glial progenitors during embryogenesis (Hartfuss et al., 2001; Than-Trong and Bally-Cuif, 2015). The expression of these specific radial glial markers revealed a delay in their onset of expression in the rhombomere centers with respect to *sox9b*. Expression of *fabp7a* was first observed at 30 hpf, restricted to discrete groups of cells located mediolaterally all along the AP axis (Figure 1G). Later, *fabp7a* expression became enriched within stripes (Figures 1H–J), which corresponded to the rhombomere centers (Figures 1K, K’), until at least 54 hpf. Similarly, the onset of *slc1a2a* expression was between 30 and 39 hpf (Figures 1L, M), and the enrichment was sustained until 54 hpf (Figures 1M–O), a period when most of the cells within the adjacent regions had already engaged in neurogenesis (Gonzalez-Quevedo et al., 2010). Furthermore, we confirmed that both *fabp7a* and *slc1a2a* were expressed in the same cells within the rhombomere centers (Figures 1P,P’). Overall, the spatiotemporal analysis of progenitor cell markers within the rhombomeres reveals that rhombomere centers show a specific combination of gene expression with the enrichment of some radial glial markers.

### The centers of the rhombomeres harbor non-committed and actively proliferating progenitor cells

**FIGURE 2:**
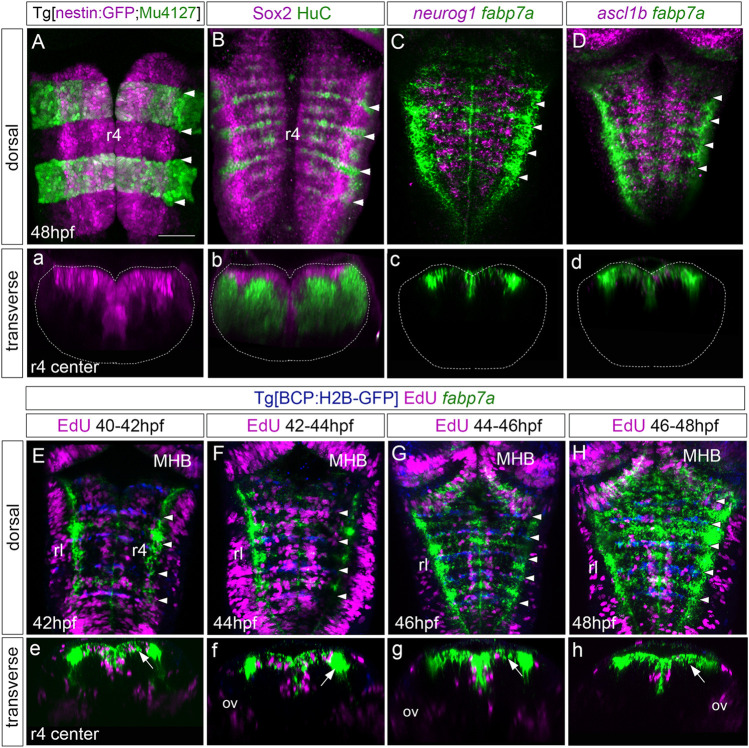
Rhombomere centers harbor proliferating neural progenitors. **(A)** Tg[nestin:GFP; Mu4127] embryos at 48 hpf displaying neural progenitors in magenta and the r3 and r5 landmarks in green. **(B)** Wild-type embryos immunostained with anti-Sox2 (magenta) to visualize the neural progenitors and with anti-HuC (green) to label the differentiated neurons at 48 hpf. **(C,D)** Wild-type embryos *in situ* hybridized with *fabp7a* (green) to stain progenitors and *neurog1* or *ascl1b* (magenta) to label neuronal committed cells at 48 hpf. **(a–d)** Transverse views of **(A–D)** through the center of r4. **(a)** Images displaying a single channel or **(b–d)** the overlay of both channels. **(E–H)** Tg[BCP:H2B-GFP] embryos were incubated with EdU for 2h, *in situ* hybridized with *fabp7a*, and immunostained with anti-GFP to label boundary cells. The EdU-positive cells are displayed in magenta, the boundaries in blue, and *fabp7a* expression indicating the center of the rhombomeres in green. **(e–h)** Transverse views of **(E–H)** through the r4 center. White arrows in **(e–h)** indicate *fabp7a* cells that did not incorporate EdU. **(A–H)** Dorsal MIP with anterior to the top displaying all channels. White arrowheads indicate the rhombomeric boundaries. Dotted lines in **(a–d)** indicate the contour of the neural tube. ov, otic vesicle; rl, rhombic lip. Scale bar, 50 μm.

Next, we sought to investigate the type of progenitors present in the rhombomere centers. We first analyzed the expression of neural markers such as nestin and Sox2, which are shared between neuroepithelial cells and radial glial progenitors. We observed that nestin was expressed in the hindbrain ventricular zone without any spatial restriction along the AP axis (Figures 2A, a). Accordingly, Sox2—another pan-neural progenitor marker—was expressed in the ventricular domain all along the AP axis and did not overlap with HuC, a pan-neuronal differentiation marker expressed in the neuronal differentiation domain (Figures 2B, b). To map the asynchronous patterning of neurogenesis within individual rhombomeres, we performed a colocalization analysis of *fabp7a* and the proneural genes *neurog1* and *ascl1b*, which label cells committed to the neuronal lineage (Bertrand et al., 2002). Double *in situ* hybridization experiments revealed that cells in the center of the rhombomeres (*fabp7a* cells) did not display *neurog1* and *ascl1b* expression, which were restricted to the neighboring domains (Figures 2C, D), the boundary-flanking regions (Nikolaou et al., 2009), and in a more ventral cell layer corresponding to the neurogenic committed regions (Belzunce et al., 2020; Hevia et al., 2022). Accordingly, *fabp7a* was expressed in the ventricular progenitor cells (Figures 2c, d). These observations indicate that at this stage (48hpf), cells within rhombomere centers are non-committed progenitors, whereas progenitors in the boundary-flanking regions had already engaged in neurogenesis (Gonzalez-Quevedo et al., 2010).

With the increasing evidence that cells within rhombomere centers display distinct features from those in the neighboring regions, we wanted to seek whether these molecular differences translated into distinct proliferative behaviors. First, we studied the putative changes in proliferative activity by EdU incorporation experiments. To reveal which cells underwent DNA replication, we shortly pulsed Tg[BCP:H2B-GFP] embryos displaying GFP in the hindbrain boundaries (Hevia et al., 2022) with EdU at different time intervals. This was followed by *fabp7a* staining to label the cells in the rhombomere centers (Figures 2E–H, e–h). We observed that during 40–42 hpf and 42–44 hpf intervals, many cells in the whole hindbrain incorporated EdU, including the mid-hindbrain boundary (MHB) and the rhombic lip (rl) (Figures 2E, F, e, f), suggesting that cells at the center of the rhombomere underwent S-phase during these periods. However, despite many *fabp7a*-cells still proliferating during the 44–46 hpf period (Figures 2G, g), the overall hindbrain progenitors’ proliferative capacity diminished (Supplementary Figure S1). During the 46–48-hpf period, almost no cells within the rhombomere centers had incorporated EdU (Figures 2H, h; see white arrows), except for some cells in the more medioventral domain (Figures 2g, h), suggesting that the derivatives of rhombomere centers might be neurons. Accordingly, upon cell quantification, we observed that the number of *fabp7a* cells within the rhombomere centers that incorporated EdU decreased over time (Supplementary Figure S1). These results indicate that the centers of the rhombomeres harbor proliferating progenitors that diminish their proliferative capacity from 46 hpf onward.

### The progenitor cells within the rhombomeric centers are maintained in the G1-phase

**FIGURE 3:**
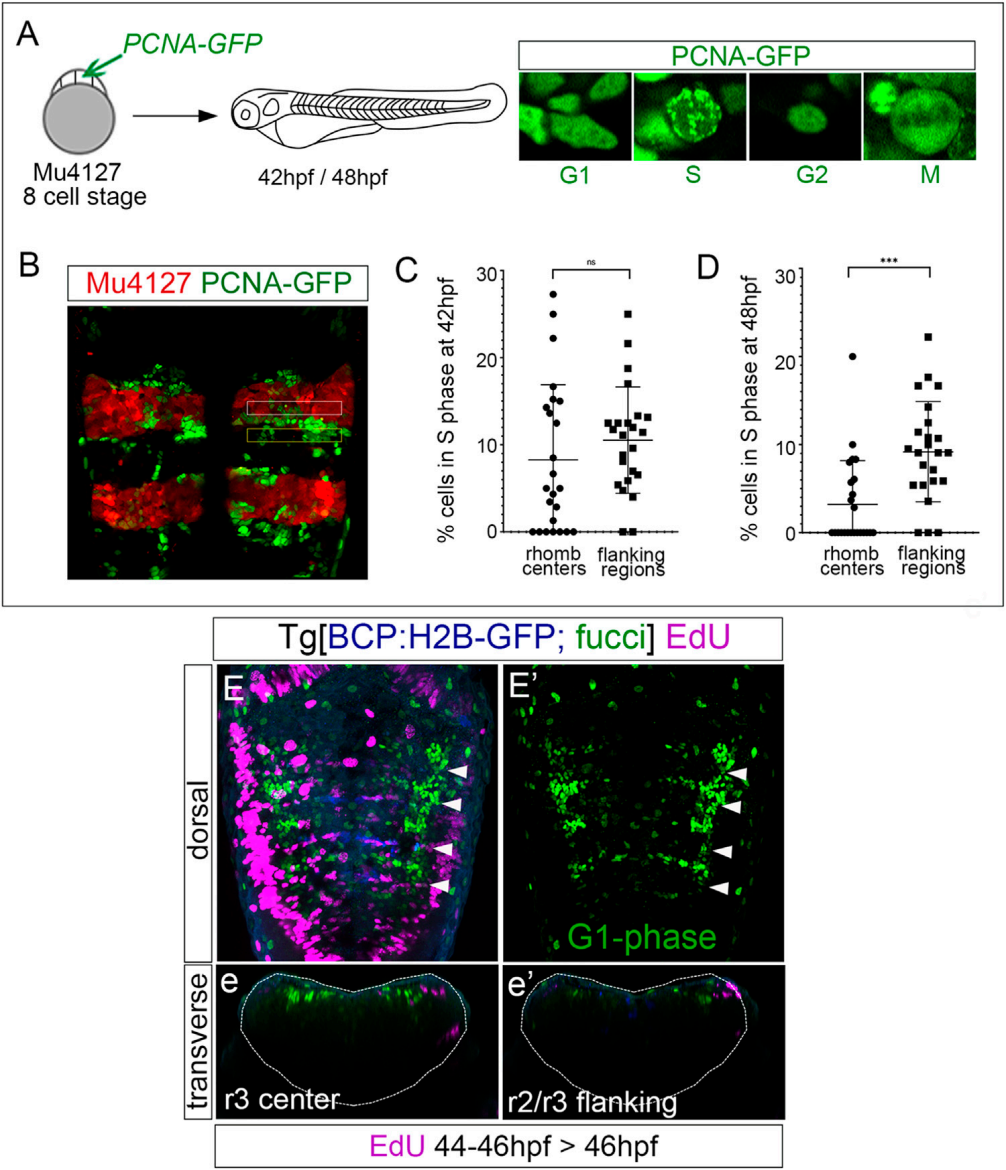
Rhombomere centers harbor G1-phase-arrested progenitors. **(A)** Scheme depicting the experimental design of the *in vivo* PCNA-GFP clonal analysis. Mu4127 embryos displaying mCherry in r3 and r5 were injected with PCNA-GFP at the 8-cell stage, and hindbrains were analyzed at 42 hpf and 48 hpf. Images show examples of the different distributions of PCNA-GFP within the cell nuclei along the cell cycle phases. **(B)** Example of the hindbrain of Mu4127 embryos injected with PCNA-GFP used for quantification analysis. Rhombomere centers and boundary-flanking regions used for quantification are framed in white and yellow, respectively. **(C,D)** Graphs illustrating the percentage of cells in the S-phase in rhombomere centers and boundary-flanking regions at 42 hpf and 48 hpf, respectively. S-phase cells at 42 hpf: 8.2% in rhombomere centers vs. 10.5% in boundary-flanking regions. S-phase cells at 48 hpf: 3.2% in rhombomere centers vs. 9.1% in boundary-flanking regions. Wilcoxon test analysis is shown: ns, non-significant, ***p < 0.001, N = 4 embryos, n = 24 boundaries, n = 24 flanking regions. **(E,E’)** Double transgenic Tg[BCP:H2B-GFP; fucci] embryos were incubated with EdU for 2 h and analyzed at 46 hpf. Boundaries are depicted in blue, G1-phase cells in green, and EdU-positive cells in magenta. Note that G1-phase cells are mainly located in the center of the rhombomeres and did not incorporate EdU. Dorsal MIP with anterior to the top displaying a merge of channels **(E)** and only G1-phase cells in the green channel **(E’)**. White arrowheads indicate the rhombomere boundaries. **(e,e’)** Transverse views of **(E)** at the level of the r3 center and r2/r3 flanking region, respectively. Dotted lines in **(e,e’)** indicate the contour of the neural tube.

Next, we explored whether progenitor cells that decreased the proliferative capacity were arrested in a specific cell cycle phase. We performed an *in vivo* analysis of PCNA-GFP dynamics by injecting it in Mu4127 embryos, expressing mCherry in rhombomeres 3 and 5. We imaged PCNA-GFP before and after these cells decreased the proliferative activity, at 42 hpf and 48 hpf, respectively, and focused on the cell nuclei PCNA-GFP localization patterns within the rhombomere centers and the adjacent flanking regions. We paid particular attention to cell nuclei displaying a finely speckled signal as a mark of the S-phase (Figures 3A, B). Quantitative analysis at 42 hpf showed that both rhombomere centers and the adjacent flanking regions contained a similar percentage of cells in the S-phase (Figure 3C; 8.2% in rhombomeres’ centers vs. 10.5% in flanking regions). However, at 48 hpf, the S-phase cells in the rhombomere centers decreased significantly (Figure 3D; 3.2% in rhombomeres’ centers vs. 9.1% in flanking regions). This evidence lets us further explore the possibility that these progenitors could be arrested in the cell cycle. For this, we made use of the Tg[Fucci] line (Bouldin and Kimelman, 2014), where cells in the G1-phase display mCherry-zCdt1. We pulsed embryos with EdU at 46 hpf for 2 h—the time when cell proliferation started to decrease—and imaged them to assess the colocalization of EdU-positive cells (S-phase cells) and fucci cells (G1-phase cells) within the hindbrain. G1-phase cells were enriched in the center of the rhombomeres, with a pattern very similar to that of *fabp7a* (Figures 3E, E′), and, as expected, EdU-positive cells did not display the G1-phase marker (Figures 3e, e’). These pieces of evidence suggest that from 46 hpf onward, rhombomere centers harbor cells that may remain as quiescent progenitors.

### Proliferative progenitors in rhombomere centers exhibit different division modes

**FIGURE 4:**
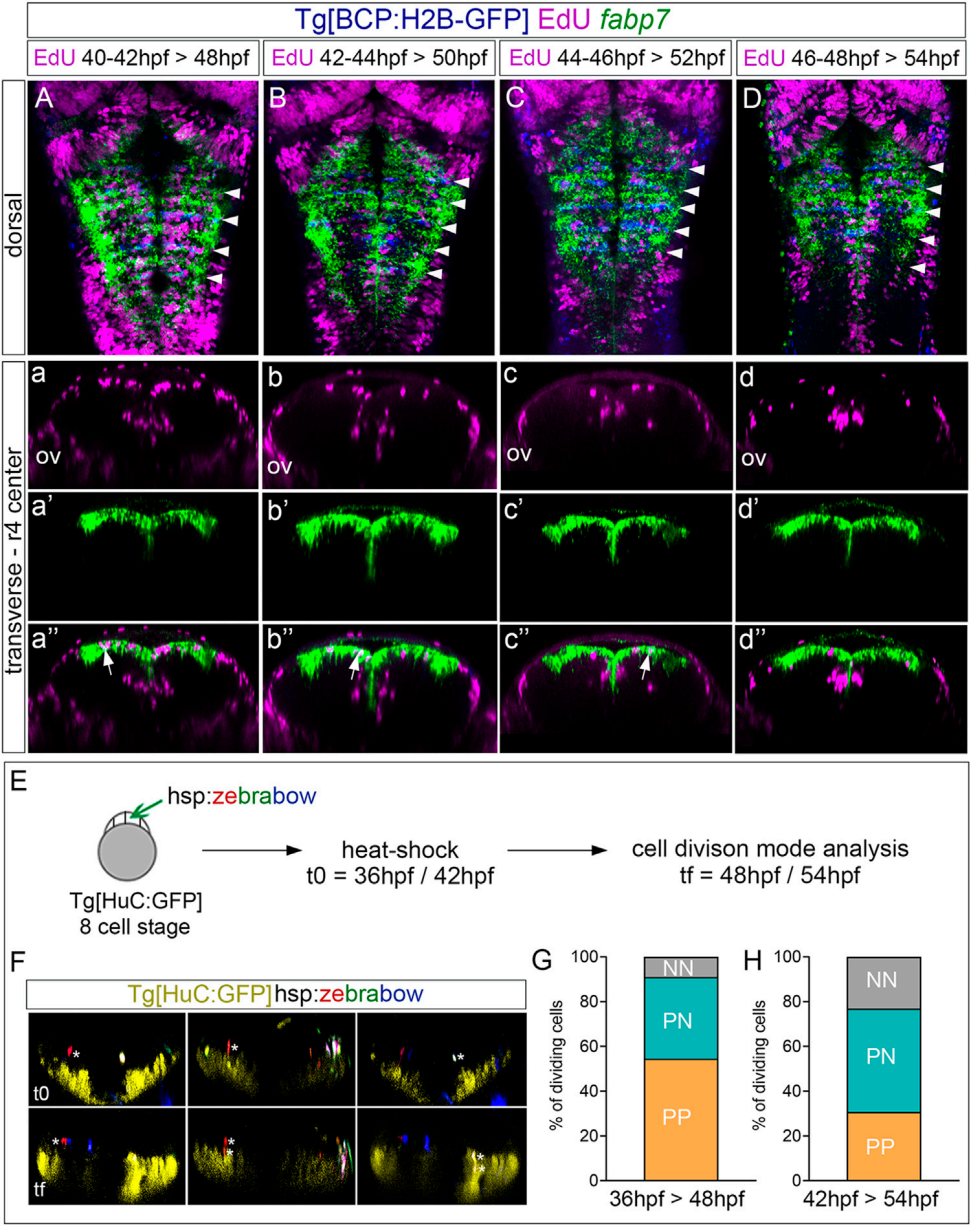
Proliferating progenitor cells within the rhombomere centers shift their division mode over time. **(A–D)** Tg[BCP:H2B-GFP] embryos were pulsed with EdU at the indicated intervals and chased for 6 h to analyze the position of the derivatives of cells that underwent the S-phase. EdU is depicted in magenta, hindbrain boundaries in blue, and *fabp7a* cells enriched in the center of the rhombomeres in green. **(A–D)** Dorsal projections with anterior to the top. White arrowheads indicate the rhombomere boundaries. **(a–d, a’–d’, a’’–d’’)** Transverse views of **(A–D)** through the center of r4 displaying either the EdU cells **(a–d),** the *fabp7a* cells **(a’–d’)**, or the merge of both **(a’’–d’’)**. ov, otic vesicle. **(E)** Analysis of the progenitor cell division mode within the rhombomere centers. Scheme depicting the multicolor clonal growth experimental approach. Tg[HuC:GFP] embryos at the 8-cell stage were injected with the hsp:zebrabow construct and heat-shocked 1 h before the imaging time. Embryos were *in vivo* imaged at 36 hpf or 42 hpf (t0), and then at 48 hpf or 54 hpf (tf), respectively. The cell division mode was analyzed in all the clones. **(F)** Transverse projections generated at t0 and tf showing examples of the three observed clonal cell behaviors: a cell undergoing symmetric proliferative division (PP, red cell not expressing HuC at t0 and the daughter cells not expressing it at tf; white asterisks), a cell undergoing asymmetric division (PN, red cell not expressing HuC at t0, with only one of the daughters expressing HuC at tf; white asterisks), and a cell undergoing symmetric neurogenic division (NN, white cell not expressing HuC at t0, but both daughters expressing HuC at tf; white asterisks). The neuronal differentiation domain is displayed in yellow. **(G,H)** Stacked bar graphs showing the percentage of PP (orange), PN (blue), and NN (gray) cell division modes in the rhombomere centers between two different time intervals: 36–48 hpf (G; n = 11 cells, N = 4 embryos) and 42–54 hpf (H; n = 13 cells N = 5 embryos).

Next, to assess the derivatives of the progenitors at the rhombomere centers, we performed EdU-pulse-and-chase experiments, which allowed us to analyze the final position of the cells that incorporated EdU at the given pulse time. Thus, Tg[BCP:H2B-GFP] embryos after a 2 h EdU-pulse were chased for 6 h and *in situ* hybridized with *fabp7a*. Then, the position of EdU-positive cells in the ventricular vs. neuronal differentiated domain was assessed (Figures 4A–D, a–d, a’–d’, a’’–d’’). Although at 48 hpf, most of the EdU-positive cells remained in the ventricular progenitor zone and expressed *fabp7a*, and some were found in the neuronal differentiation domain (Figures 4a–a’’). In line with our previous results, the *fabp7a* cells in the S-phase diminished over time (Figures 4B, C, b, c, b’, c’, b’’, and c’’), with a dramatic decrease of cells that incorporated EdU from 46 hpf onward (Figures 4D, d, d’’). Thus, these results suggested that progenitor cells within rhombomere centers can give rise to neuronal derivatives. However, we could not rule out if the remaining ventricular progenitors were maintained as quiescent progenitors out of the cell cycle or if they were slow-dividing progenitors holding longer cell cycles that last more than 8 h, described as the estimated average time for hindbrain cells to undergo division (Lyons et al., 2003).

Since progenitor cells within the rhombomere centers seem to contribute to the neuronal lineage, the next step was to unveil which was the cell division mode. For this, we used the Zebrabow multicolor clonal analysis approach (Brockway et al., 2019), which allows us to label in the same color all the derivatives from a given cell clone. We classified the different division modes according to two criteria: the relative position between sister cells after division and the expression of the HuC neuronal differentiation marker. Tg[HuC:GFP] embryos at the 8-cell-stage were injected with the hsp:zebrabow construct, heat-shocked at two different times (36 hpf or 42 hpf), imaged, and let to develop for 12 more hours before imaging again (Figure 4E). Then, the percentage of cells in the rhombomere centers undergoing the different division modes was assessed: symmetric proliferative, giving rise to two progenitor cells (PP); asymmetric, giving rise to one progenitor and one neuronal derivative (PN); and symmetric neurogenic, giving rise to two neurons (NN) (Figure 4F; see one example of each mode of cell division). Although, at 48 hpf, most cell divisions were symmetric proliferative (Figure 4G; PP: 54.5% vs. PN: 36.3% vs. NN: 9.2%), by 54 hpf, the asymmetric and neurogenic divisions increased at the expense of the proliferative divisions (Figure 4H; PP: 30.7% vs. PN: 46.2% vs. NN: 23.1%), supporting the change in behavior of these cells that we previously envisaged.

### Progenitor cells in the rhombomeric centers display Notch activity

**FIGURE 5:**
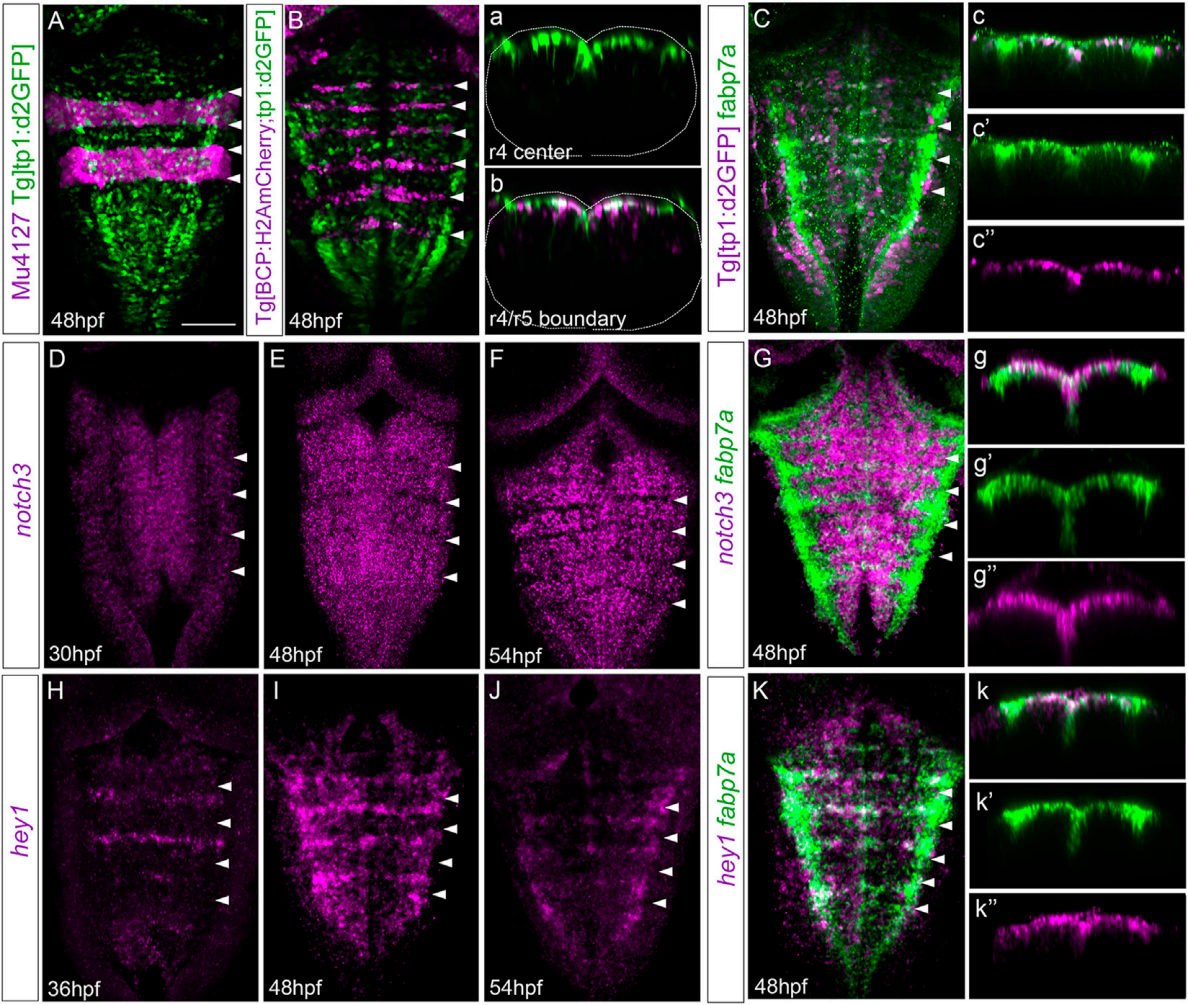
Rhombomere centers display Notch activity. **(A,B)** Mu4127 Tg[tp1:d2GFP] and Tg[BCP:H2AmCherry; tp1:d2GFP] embryos as the readout of Notch activity (green) with r3 and r5 or boundaries (magenta) as landmarks at 48 hpf. **(a,b)** Transverse views of **(A,B)** through the center of r4 or the r4/r5 boundary. Dotted lines indicate the contour of the neural tube. (C) Tg[tp1:d2GFP] embryos displaying Notch activity (magenta) and immunostained with fabp7a (green) at 48 hpf. **(c–c’’)** Transverse view of **(C)** showing only the ventricular domain through the center of r4 displaying either both channels **(c)** or single channels **(c’–c’’)**. **(D–G)** Wild-type embryos *in situ* hybridized either with *notch3* **(D–F)** or *notch3* and *fabp7a* **(G)** at the indicated stages. **(g–g’’)** Transverse view of **(G)** showing only the ventricular domain through the center of r4 displaying either both channels **(g)** or single channels **(g’–g’’)**. **(H–K)** Wild-type embryos were *in situ* hybridized either with *hey1* **(H–J)** or *hey1* and *fabp7a* **(K)** at the indicated stages. **(k–k’’)** Transverse view of **(K)** showing only the ventricular domain through the center of r4 displaying either both channels (k) or *fabp7a* or *hey1* **(k’–k’’)**. **(A–K)** Dorsal MIP with anterior to the top. White arrowheads indicate the rhombomere boundaries. Scale bar, 50 μm.

We were eager to understand by which mechanism cells within the center of the rhombomeres behave differently than their adjacent neighbors. Since we previously showed that Notch signaling was important in governing the regulation of binary cell choices in the hindbrain boundaries (Hevia et al., 2022), we investigated whether Notch activity played any role in the rhombomere centers. First, we assessed whether Notch activity was spatiotemporally restricted within the hindbrain at these late embryonic stages using a readout of Notch-active cells, the Tg[tp1:d2GFP] line (Clark et al., 2012). At 24 hpf, rhombomeres behave as proneural clusters (Nikolaou et al., 2009), and therefore, Notch activity is distributed in the whole segment (Hevia et al., 2022). However, by 48 hpf, there was an enrichment of Notch activity within the center of the rhombomeres (Figures 5A, B) and in the hindbrain boundaries (Figure 5B; (Hevia et al., 2022). Notch activity was confined to progenitor cells, which displayed radial projections toward the mantle zone (Figures 5a, b; Hevia et al., 2022). To demonstrate that *fabp7a* cells were Notch-active, we labeled Tg[tp1:d2GFP] embryos with fabp7a and revealed that most of the fabp7a cells in the rhombomere centers displayed Notch activity (Figures 5C, c, c’’). These results indicated that progenitor cells within the rhombomeric centers that are Notch-active display an enrichment of *fapb7a* and *slc1a2a* expression. Next, we analyzed the expression of Notch signaling players within the hindbrain. First, we studied the expression of Notch receptors, focusing on *notch3*, since it was described to be crucial in maintaining progenitor cells within a non-committed progenitor state (Than-Trong et al., 2018; Hevia et al., 2022). The spatiotemporal analysis of *notch3* revealed its faint expression in the hindbrain progenitor domain by 30 hpf (Figure 5D; (Hevia et al., 2022), although it increased by 36 hpf and was maintained at least until 54 hpf (Figures 5E, F). Colocalization analysis at 48 hpf revealed the overlapping expression of *notch3* and *fabp7a* in the rhombomere centers (Figures 5G, g–g’’), supporting the idea that *notch3* might maintain this cell population in a non-committed progenitor state. However, both *notch1a* and *notch1b* were equally well-expressed in the rhombomeric centers and overlapped with *fabp7a* (Supplementary Figure S2). To further explore the complex Notch network within this cell population, we investigated the expression of the Notch signaling targets. Previous work in adult zebrafish pallium described that quiescent radial glial cells expressing *fabp7a* maintain their stemness features upon Notch3 signaling by the target gene *hey1* (Than-Trong et al., 2018). Moreover, in the zebrafish retina, *hey1* regulates the Muller glia downstream *notch3*, thereby reducing their proliferative capacity upon injury (Sahu et al., 2021). Thus, these lines of evidence drove us to *hey1* as a good candidate downstream of *notch3*. We performed the spatiotemporal expression analysis of *hey1* within the zebrafish hindbrain. Although at 24 hpf, no expression of *hey1* was observed in the hindbrain (result not shown), by 36 hpf, *hey1* was specifically expressed in rhombomere centers and remained at least until 54 hpf (Figures 5H–J). *hey1* overlapped with *fabp7a* at the center of the rhombomeres (Figure 5K), although not all *fabp7a* progenitors displayed *hey1* (Figures 5k–k’’), suggesting that the Notch pathway may operate through *hey1* in a subpopulation in rhombomere centers.

Notch3 is necessary for the maintenance of cells within rhombomere centers as non-committed progenitors.

**FIGURE 6:**
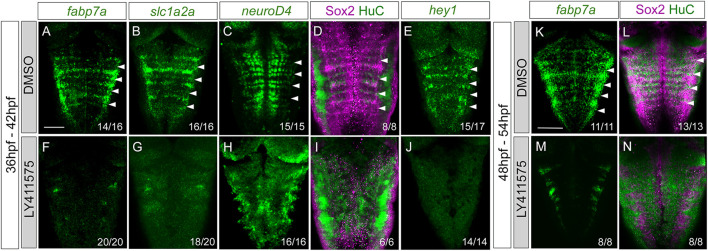
Progenitor cells in the rhombomere centers are Notch-responsive. Wild-type embryos were treated with either DMSO **(A–E, K–L)** or the gamma-secretase inhibitor LY411575 **(F–J, M,N)** for 6 h at 36 hpf (A–J) or at 48 hpf **(K–N)**. Embryos were *in situ* hybridized with *fabp7a* **(A,F,K,M)**, *slc1a2a* **(B,G)**, *neuroD4* **(C,H)**, and *hey1* **(E,J)**, or immunostained with Sox2 and HuC **(D,I,L,N)**. Dorsal MIP with anterior to the top. White arrowheads indicate the rhombomere boundaries. Numbers at the bottom indicate the individuals with the displayed phenotype over the total of analyzed specimens. Scale bar, 50 μm.

The display of Notch activity and the expression of Notch players within rhombomere centers led us to find whether these progenitors responded to Notch. For this, we conditionally abrogated Notch signaling using the pharmacological reagent LY411575, which is a gamma-secretase inhibitor resulting in the downregulation of the Notch pathway. First, we inhibited Notch activity by incubating Tg[tp1:d2GFP] embryos with either DMSO as the control (Figures 6A–E) or LY411575 (Figures 6F–J) from 36 to 42 hpf (Figures 6A–J), before rhombomere centers decrease their proliferative capacity, and analyzed the effects on progenitor cells, committed progenitors, differentiated neurons, and the Notch target *hey1*. Upon abrogation of Notch activity, the expression of *fabp7a* and *slc1a2a* progenitor markers was completely downregulated when compared to that of control embryos (Figures 6A, B, F, G). Neurogenesis and neuronal differentiation increased upon Notch inhibition, and the well-described proneural gene salt-and-pepper expression pattern was lost (Figures 6C, H), demonstrating that cells dramatically engaged in neurogenesis. Accordingly, there was an increase in the neuronal differentiation domain at the expense of the progenitor domain (Figures 6D, I). Moreover, the expression of the Notch target *hey1* was completely inhibited (Figures 6E, J), suggesting that Notch activity was acting through *hey1* in the rhombomere centers. These results indicated that Notch signaling maintains hindbrain progenitor cells—including those within rhombomere centers—as non-committed progenitors. To dissect whether the Notch pathway was important for maintaining these cells in the progenitor state at later stages, we inhibited Notch in a later temporal window, from 48 to 54 hpf, and observed a similar phenotype (Figures 6K–N). When we quantified the volume of the progenitor vs. the neuronal differentiation domains in the control and LY41575-treated embryos at different stages, we confirmed this phenotype (Supplementary Figures S3A, B) and demonstrated that it was not a result of an increase in cell death (Supplementary Figure S3C).

**FIGURE 7:**
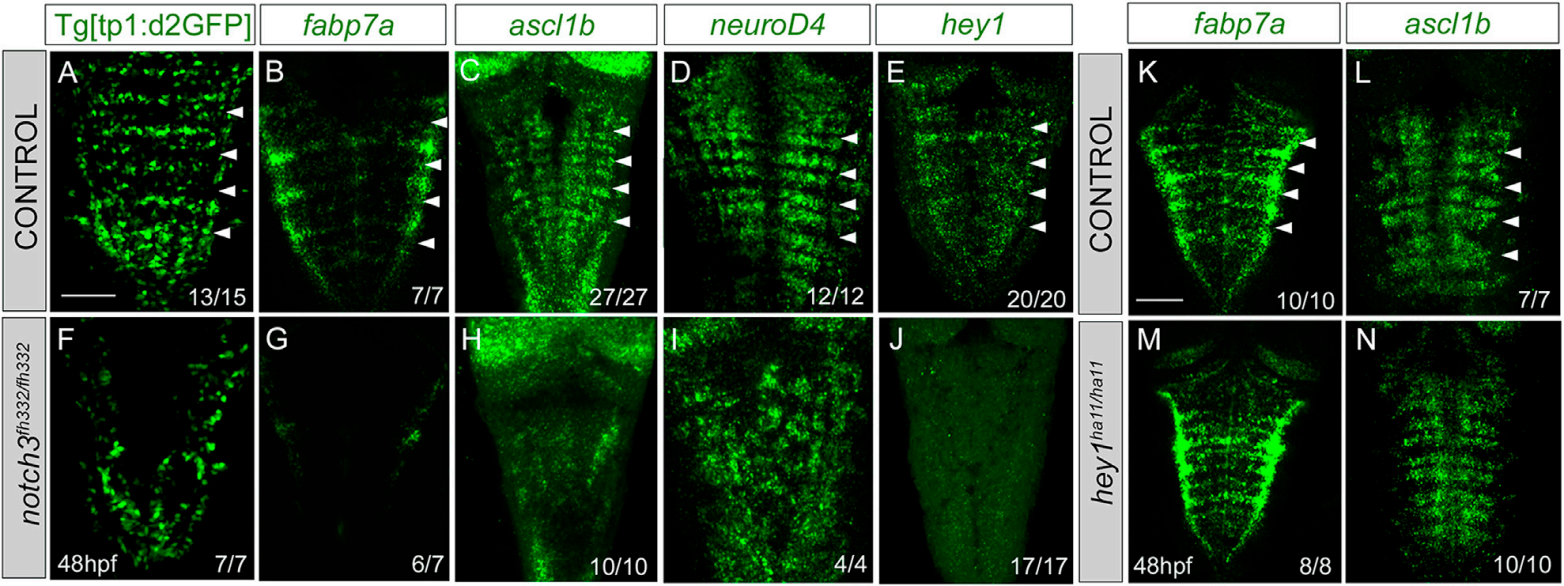
*notch3* mutation accounts for the loss of Notch activity in the center of the rhombomeres and results in neuronal differentiation. **(A–J)** Control and *notch3* ^*fh322/fh322*^ embryos in the Tg[tp1:d2GFP] background were *in situ* hybridized with *fabp7a* (B, G), *ascl1b* **(C,H)**, *neuroD4* **(D,I)**, or *hey1* **(E,J)** at 48 hpf. **(K–N)** Control and *hey1* ^*ha11/ha11*^ embryos were *in situ* hybridized with *fabp7a* **(K,M)** and *ascl1b* **(L,N)** at 48 hpf. Dorsal MIP with anterior to the top. White arrowheads indicate the rhombomere boundaries. Numbers at the bottom indicate the individuals with the displayed phenotype over the total of analyzed specimens. As control embryos, either wild-type or heterozygous embryos were used. Scale bar, 50 μm.

To study the contribution of Notch3 signaling to the Notch activity, we inhibited the expression of *notch3* by the use of the null *notch3* ^*fh332/fh332*^ mutants (Alunni et al., 2013) combined with the Notch activity reporter. Tg[tp1:d2GFP;*notch3* ^*fh332/fh332*^] embryo analysis revealed a dramatic decrease of the patterned Notch activity in the hindbrain (Figures 7A, F). Next, we analyzed whether Notch3 was involved in ascribing the progenitor capacities to this specific cell population. *notch3* mutation resulted in a loss of *fabp7a* expression in the rhombomere centers (Figures 7B, G), demonstrating the relevant contribution of Notch3 activity in the *fabp7a* progenitors. Expression of the proneural genes *ascl1b* and *neuroD4* was affected as well (Figures 7C, D, H–I), as a consequence of all progenitors undergoing neuronal differentiation. The expression of the Notch target *hey1* was also downregulated (Figures 7E–J). To account for any role of apoptosis within this phenotype, we analyzed cell death figures, and no differences in the number of apoptotic events were observed between wild type and *notch3* ^*fh332/fh332*^ mutants (wild type: 0.4 ± 0.8 apoptotic cells n = 16 vs. *notch3* ^*fh332/fh332*^ 1 ± 2.2 apoptotic cells n = 14, ns). Therefore, these results indicate that *notch3* is necessary for the maintenance of the progenitor state within this cell population. However, *fabp7a* and proneural gene expression analysis in *hey1* ^*ha11/ha11*^ mutants did not show any differences when compared with that in control embryos (K—N), suggesting that *hey1* might not be the Notch3 effector in maintaining the *fabp7a* cells as progenitors during this time window.

## Discussion

Previous studies have described that differential neurogenesis in the hindbrain is spatiotemporally regulated. During the first neurogenesis phase, rhombomeres act as proneural clusters (Nikolaou et al., 2009), whereas hindbrain boundaries are devoid of neurogenesis (Voltes et al., 2019; Hevia et al., 2022). However, later on, boundary progenitor cells transition toward neurons relying on Notch3 signaling (Hevia et al., 2022). This distinct neurogenic behavior is usually foreshadowed by different gene expressions since boundaries express a specific gene combination (Cheng et al., 2004; Letelier et al., 2018). Interestingly, differences in the neurogenic capacity are generated within the same rhombomere since neurogenesis gets restricted to the boundary-flanking regions, whereas the centers of the rhombomeres instruct the position of the neighboring neurons through FGF signaling (Gonzalez-Quevedo et al., 2010). This confinement of neurogenesis to the boundary-flanking regions is regulated by miR-9, which exerts distinct actions at different stages of progenitor commitment along the neurogenesis cascade (Coolen et al., 2012). Thus, the center of the rhombomeres works as a signaling hub, which, later on, will express radial glial cell markers and provide glial cells (Esain et al., 2010). Our results provide evidence to foresee another layer of complexity in the attribution of different progenitor behaviors and stress the importance of the spatiotemporal coordination of neurogenesis. The enrichment in *sox9, meteorin*, and *meteorin-like* in the rhombomere centers during a short period of time, just before the onset of radial glial cell markers, could keep this cell population out of the early neurogenic program that the neighboring cells are already engaged in. This occurs at the same time that FGF signaling from neurons in the ventral hindbrain is required to downregulate proneural gene expression (Gonzalez-Quevedo et al., 2010). Thus, it seems that FGF signaling represses proneural genes at the centers, permitting *fabp7a*-cells to proliferate.

One strategy to properly organize the generation of the neuronal circuits is to restrict the progenitor capacities within specific territories and to maintain groups of long-lasting progenitors (for review, see Belmonte-Mateos and Pujades, 2022). Several examples of the existence of distinct progenitor pools during embryogenesis (for review, see Stigloher et al., 2008) and the maintenance of quiescent cells in adult organisms (Than-Trong and Bally-Cuif, 2015; Than-Trong et al., 2018; Urbán et al., 2019) have been described. Our data show that this could also occur in the hindbrain. We demonstrate, for the first time, that the center of the rhombomeres harbor proliferating progenitors since embryos display EdU labeling across the hindbrain, including the rhombomeric centers. However, at a later temporal window, beyond 48 hpf, their division capacity decreases significantly and fewer cells are in the S-phase. Accordingly, we could demonstrate that beyond 46 hpf, the centers of the rhombomeres are enriched in progenitors in the G1-phase, which considering the significant decrease in proliferation at these stages, would indicate that rhombomere centers harbor progenitors in a quiescent or slow division mode. These would suggest that different strategies are used within the same tissue to differentially pattern neurogenesis, or in other words, to restrict the progenitor features. Moreover, our zebrabow clonal analysis experiments also unraveled that of those progenitors that divide, they do so in different modes. In addition, in line with our previous results, these confirm that at a later developmental window, asymmetric and symmetric neurogenic cell divisions increased at the expense of symmetric proliferative divisions. Although these clonal data support the change in the ratio of the rhombomere centers’ cell division patterns between 48 hpf and 54 hpf, the difficulty of having large numbers of clones for analysis would require further experiments with larger sample sizes to better confirm the shift in cell division. Our results depict a scenario similar to what has been found in the mouse brain, where authors demonstrated the presence of slow-dividing progenitors in the embryonic telencephalon being the precursors of quiescent neural stem cells in the adult brain (Furutachi et al., 2015; Harada et al., 2021). It is, therefore, possible that cells in rhombomere centers are spatiotemporally regulated in their proliferative and neurogenic capacity to ensure the maintenance of progenitors in order to provide neurons at a later temporal window to maintain the homeostasis of the hindbrain.

What is the molecular mechanism responsible for the neurogenic patterning within the rhombomeres? Despite FGF20 being involved in the downregulation of proneural genes in rhombomere centers (Gonzalez-Quevedo et al., 2010), we explored the possible role of Notch signaling in this cell population for its role in the maintenance of slow-dividing/quiescent cells in the zebrafish pallium (Than-Trong et al., 2018). Cells in rhombomere centers are Notch-active from 48 hpf onward, and Notch signaling maintains them as non-committed progenitors. Although other Notch receptors are expressed earlier, Notch3 onset of expression coincides with the expression of *sox9b*, *meteorin*, and *meteorin-like* in the centers and also co-expresses with its target *hey1*. Thus, it seems that Notch3 is the main Notch receptor responsible for Notch activity during later neurogenesis in the hindbrain (Hevia et al., 2022). Interestingly, it was described that the sustained expression of the Notch3 target hey1 maintained neural progenitors in a quiescent and slow-dividing mode in both adult zebrafish telencephalon and the subventricular zone in mouse embryonic brain, respectively (Furutachi et al., 2015; Than-Trong et al., 2018; Harada et al., 2021). Despite our functional studies demonstrating that cells in rhombomere centers rely on Notch3 to remain non-committed progenitors, they also suggest that *hey1* might not be the target through which it exercises its function within hindbrain rhombomeres. Most probably, the scenario is more complex, and other *hey* or *her* genes could compensate for the effects of abrogating *hey1* as a way of providing robustness to the system.

## Data Availability

The raw data supporting the conclusion of this article will be made available by the authors, without undue reservation.

## Supplementary material

SUPPLEMENTARY FIGURE S1 Proliferative activity of the fabp7a cells in rhombomere centers decreases over time. Dot-plot displaying the number of fabp7a cells that incorporated EdU at the given time interval in rhombomere centers. Each dot corresponds to a rhombomere center [40–42 hpf: 21.9 cells (SD 2.5, SEM 0.6); 42–44 hpf: 12.4 cells (SD 1.9, SEM 0.5); 44–46 hpf: 8.6 cells (SD 2.9, SEM 0.7); 46–48 hpf: 1.9 cells (SD 1.6, SEM 0.4); p < 0.0001 (***)]. Multiple comparisons using one-way ANOVA with Tukey’s multiple comparison test among all groups were performed; n = 16 rhombomere centers, N = 4 embryos, with 4 rhombomere centers per embryo.

SUPPLEMENTARY FIGURE S2 notch1a and notch1b receptors are expressed all along the AP axis. **(A,B)** Embryos were *in situ* hybridized with fabp7a (green) and notch1a or notch1b (magenta) at 48 hpf. Dorsal MIP with anterior to the top. White arrowheads indicate the rhombomere boundaries. **(a,b)** Transverse views of **(A,B)** through the center of rhombomere 4.

SUPPLEMENTARY FIGURE S3 Neuronal differentiation domain increases at the expense of the progenitor domain in the rhombomeres upon inhibition of the Notch pathway. **(A,C)** Dot-plots showing the volume of the Sox2 progenitor domain **(A)**, the HuC differentiated domain **(B)** in r4, and the total hindbrain apoptotic events **(C)** in embryos treated with DMSO or LY411575 at the indicated intervals. Each dot corresponds to an embryo. **(A)** Sox2 volumes of r4 in embryos treated between 36 and 42 hpf were DMSO 25.1 ± 4.3 n=8 vs. LY411575 1.5 ± 1 n=6, p<0.0005 and in embryos treated between 48 and 54 hpf were DMSO 23.7 ± 5.3, n=8 vs. LY411575 10 ± 4.1 n=8, p<0.001. **(B)** HuC volumes of r4 in embryos treated between 36 and 42 hpf were DMSO 76.4 ± 3.6 n=8 vs. LY411575 98.5 ± 1 n=6, p<0.0005 and in embryos treated between 48 and 54 hpf were DMSO 75.3 ± 5.3 n=8 vs. LY411575 90 ± 4.1 n=8, p<0.0005. Note the increase in the neuronal differentiation domain accompanied with the decrease in the progenitor domain upon Notch inhibition. **(C)** Apoptotic events in embryos treated between 36 and 42 hpf were DMSO 0.3 ± 0.9 n=17 vs. LY411575 0.8 ± 1.5 n=19, ns and in embryos treated between 48 and 54 hpf were DMSO 0.25 ± 0.6 n=16 vs. LY411575 1.1 ± 2.1 n=17, ns. In all cases, the non-parametric Mann–Whitney test was used.
