# Supplementary figures and images for: Hindbrain rhombomere centers harbor a heterogenous population of dividing progenitors which rely on Notch signaling
Source: Front Cell Dev Biol. 2023 Nov 2;11:1268631. doi: 10.3389/fcell.2023.1268631 (PMC10652760; doi:10.3389/fcell.2023.1268631)

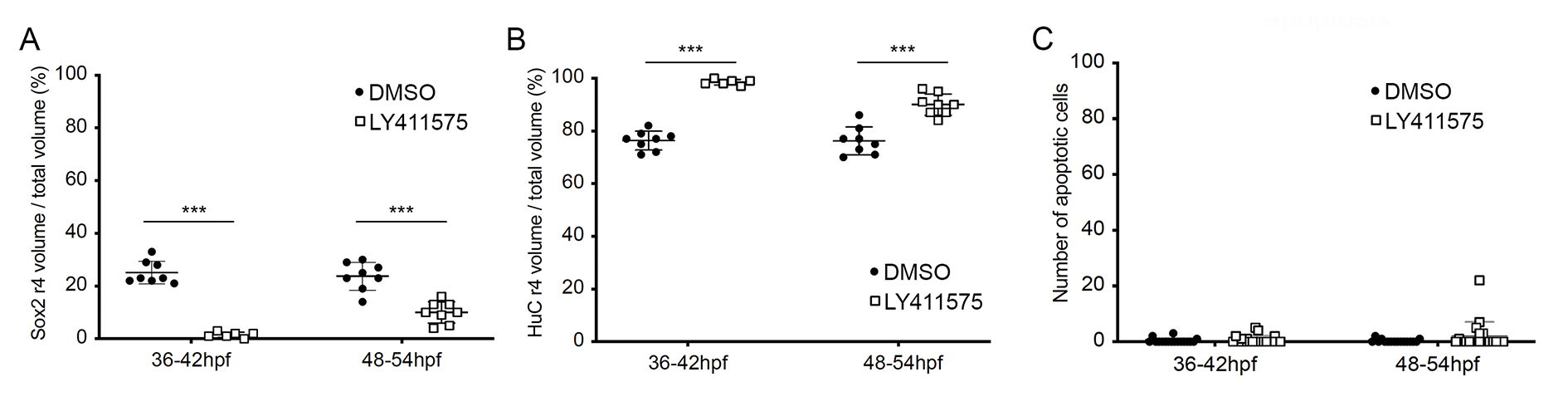

Supplement: Supplementary file 1 [file Image3.tif]

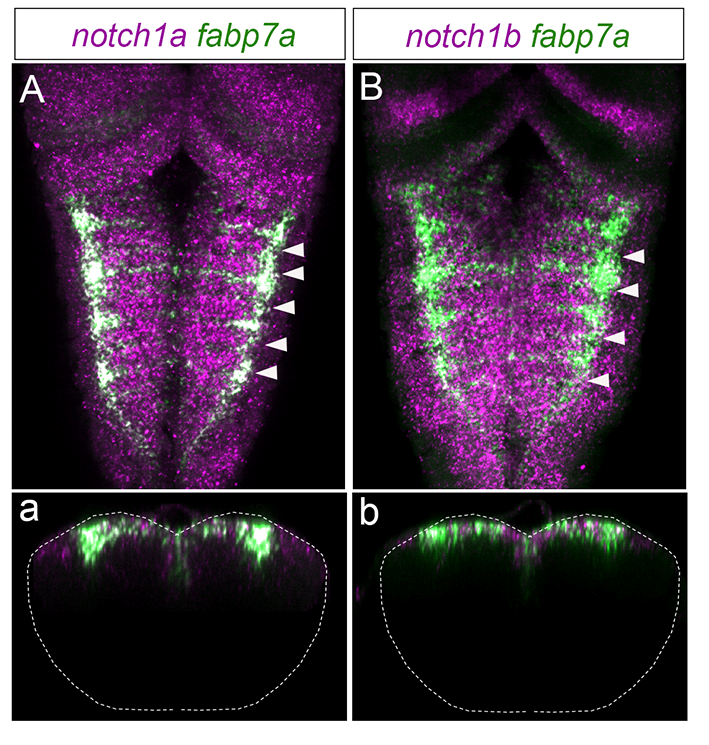

Supplement: Supplementary file 2 [file Image2.tif]

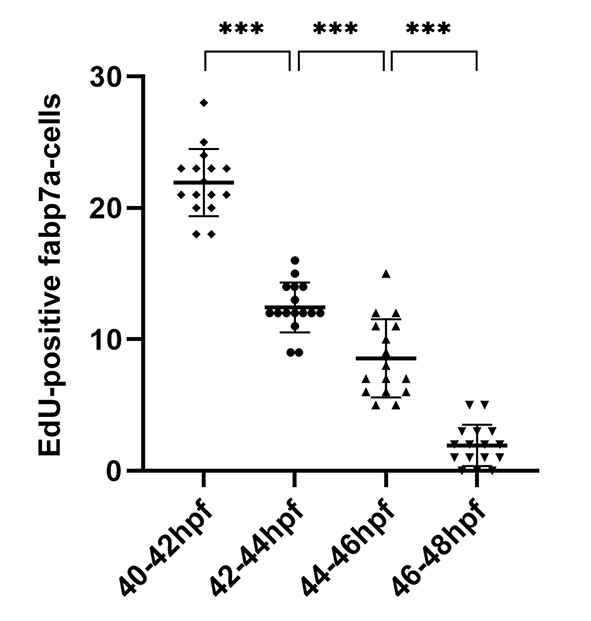

Supplement: Supplementary file 3 [file Image1.tif]
